# The impact of contract service policy and doctor communication skills on rural patient-doctor trust relationship in the village clinics of three counties

**DOI:** 10.1186/s12913-019-3875-x

**Published:** 2019-03-22

**Authors:** Linni Gu, Jianjun Deng, Huiwen Xu, Shengfa Zhang, Min Gao, Zhiyong Qu, Weijun Zhang, Donghua Tian

**Affiliations:** 1 School of Social Development and Public Policy, Beijing Normal University, 19 Xinjiekou Wai Street, Haidian District, Beijing, 100875 China; 2 Institute of Developmental Psychology, Beijing Normal University, 19 Xinjiekou Wai Street, Haidian District, Beijing, 100875 China; 3 Department of Public Health Sciences, University of Rochester School of Medicine & Dentistry, Rochester, NY 14642 USA

**Keywords:** Village doctors, Contract service, Communication, Trust, China

## Abstract

**Background:**

Trust is regarded as the cornerstone of the doctor-patient relationship in the world of medicine; it determines the decisions patients make when choosing doctors and influences patients’ compliance with recommended treatments. In China, patient-doctor trust acts as a thermometer measuring harmony in the doctor-patient relationship. The objective of this study is to explore the relationship between the contract service and patient-doctor trust-building in 25 village clinics of rural China.

**Method:**

The research was carried out in village clinics in rural China. A simple random sampling method was used to choose clinics and subjects. Based on feasibility and financial support, we chose three counties as our study settings: Dafeng District, Jiangsu Province; Yinan County, Shandong Province; and Wufeng Tujia Autonomous County, Hubei Province. Twenty-five village clinics and 574 subjects were selected in the three areas from the contract service and patient list. Descriptive statistics, *t*-tests, MANOVA, SEM, and multiple regression statistical analysis were employed to analyze the data.

**Result:**

Statistical analysis showed that contract service directly and indirectly influenced patient-doctor trust-building in village clinics. The patient perception of doctor communication skills was a mediator in the relationship between contract service policy and patient-doctor trust-building.

**Conclusions:**

Building patient-doctor trust is important in developing and enhancing rural health. The policy of contract service plays a significant role in building relationships. Well-developed communication skills of doctors contribute to the implementation of the contract service policy and to establishing patient-doctor trust.

## Background

The doctor-patient relationship in rural China plays a pivotal role in the country’s three-tiered healthcare delivery system. It is regarded as the main selection criterion used by patients to choose doctors. It has also been viewed as a key determinant of the success of village clinics in carrying out their function as the bottom tier of the three-tiered healthcare network [1]. However, the doctor-patient relationship has deteriorated since the implementation of rural health reforms in the 1980s [2]. Many studies have pointed out that the main cause of this deterioration is the degeneration of patient-doctor trust [3–6]. Trust is regarded as the keystone of the doctor-patient relationship [7] in the treatment process. It has been linked to patient satisfaction, treatment adherence, and treatment outcomes, as well as fewer malpractice lawsuits [8]. Generally speaking, a patient who has a high level of trust in his or her doctor is more likely to share relevant information, improving the outcome of treatment [7]. In view of this, China’s Health Department enacted a series of policies to improve the doctor-patient relationship in the primary health system. The most important policy for improving the relationship is the newly implemented contract service in primary health. The policy in rural China may be regarded as a Chinese counterpart of family medicine in western countries [9–12]. It can be defined as a system of contracts signed between village doctors and villagers in household units that prompts village doctors to provide demand-tailored public health services like general practitioners [13]. This policy was piloted in 15 counties in 2013 and then was implemented across the whole country in 2014. The model relies on the voluntary participation of patients and is intended to be responsive to their needs [13]. The initial purpose of enacting the policy is to increase village doctors’ annual income and provide positive incentives; however, after the policy had been deeply implemented, health researchers and managers found that the doctor-patient relationship in rural areas had changed. They considered the policy as being able to closely connect patients and village doctors, and it seemed the best way to keep patients in the primary health service. A body of research in China pointed out that implementing the contract service policy in rural area could enhance the trust of patients in village doctors [14–16]. However, as these studies used the method of normative analysis, whether the contract service has influenced rural patient-doctor trust is still unstudied empirically. Therefore, this study will analyze whether the contract service influences patient-doctor trust-building in the village clinics of rural China.

Numerous studies have shown that communication is the most effective vehicle for engendering trust [7, 17–19]. One study has found a correlation between effective patient-doctor communication and lower blood pressure, reduced anxiety, and a higher quality of life among breast cancer patients [20]. A follow-up study demonstrated that patients’ perceptions of the therapeutic communication skills of their GPs affects adherence [21]. Other studies have shown that features of doctor-patient communication can predict health outcomes [20, 22]. A more recent study analyzed a group of elderly people in the United States, showing that effective communication generates high levels of trust among elderly Medicare beneficiaries [17]. Another newly published research explored the mediating role of patient-provider communication among team members within the Patient-Centered Medical Home and patient satisfaction with providers [23]. Despite the fact that the current literature in patient-doctor communication is flourishing and proliferating, little research has investigated the mediating role of doctor-patient communication in establishing the patient-doctor relationship, especially in the village clinics of rural China.

Based on the above literature review and analysis, the main objective of this study was to explore whether there is a direct association between contract service and patient trust in doctors, whether doctors’ communication skills acted as a mediator, and whether both a direct and indirect association exist between contract service and patient trust in doctors in the village clinics of rural China, after controlling for sociodemographic factors, medical expenditures, and other health policy characteristics. Therefore, we hypothesized that the contract service policy positively influences the patient trust in doctors in the 25 village clinics of rural China that we studied, and that the patient perception of doctor communication skills mediates the pathway between contract service and patients’ trust in doctors.

## Methods

### Study settings and participants

Three counties were chosen as having different economic levels, geographic situations, and investigation feasibility and financial support: Dafeng District is located in the east plain of Jiangsu Province; Yinan County, geographically speaking, is upland and located in the west of Shandong Province; and Wufeng Tujia Autonomous County, Hubei Province, is located in a central mountainous area. The three counties not only present major differences in geography, but also show significant economic differences. Dafeng was the richest county of the three, with a GDP of RMB 647.48 billion in 2017 [24]; Yinan had a GDP of 262 billion Yuan in 2017 [25]; and Wufeng had a GDP of 65.49 billion Yuan in 2017 [26]. Dafeng was one of the pilot districts for the implementation of the contract service policy, and Wufeng and Yinan implemented the contract service policy after Dafeng.

A simple random sampling method was used to choose clinics and patients for this study. Twenty-five village clinics were selected at the Town Central Hospital in the three areas. About 20 subjects were chosen from the contract service list and outpatient list of one year in each village clinic. All of them were enrolled in the village clinics to help us complete the questionnaire as investigators. However, subjects with poor hearing, speaking difficulties, or mental health problems were excluded from the investigation. Ultimately, 625 subjects participated in the investigation by completing questionnaires after having provided informed consent. All participants received a towel as a token of appreciation. In the end, we received 574 completed questionnaires, for a response rate of 94.8%. Four investigators from Beijing Normal University, all of whom had received professional training, assisted in completing the investigation. Ethical approval was obtained from the Ethical Review Board of Beijing Normal University.

## Measures

### Doctor communication skills

The TCom-skills GP Scale (see Table 2 for items) was used to measure doctor communication skills as assessed by patients. The doctors were acting as general practitioners in primary healthcare in these rural areas; the one-dimensional scale has demonstrated high internal coherence reliability (Cronbach’s α = 0.92) and good test-retest reliability (intra-class correlation coefficient of 0.74) [21]. The scale consisted of 15 items with responses that ranged from 0 = never to 7 = always. We used the translation-and-back translation method to translate the scale. First, we translated the scale into Mandarin Chinese; we then invited a professional translator to translate it back. We carried out a pre-investigation in Shangnian Village, Shunyi District, Beijing, to assess the scale in the context of Chinese culture and achieved high internal coherence reliability (Cronbach’s α = 0.92). We calculated the mean score of the scale. Higher scores represented better communication skills of village doctors.

### Trust

A Chinese version of the Wake Forest Physician Trust Scale (WFPTS), a commonly used assessment tool in China, was applied to measure patient trust in doctors. This scale, a modified version of the WFPTS (see Table 3 for the items) in English, included ten items and measured two dimensions of patient trust in doctors. Each item was scored using a 5-point Likert scale. The reliability of the scale was shown by a value of Cronbach’s α of 0.89 in a previous study [27].

### Contract service

Contract service was measured with the following questions: Did you sign up with the contract service with village doctors? Answers were dichotomized as No = “0” and Yes = “1.”

### Patient characteristics

A number of demographic and economic variables were measured, including gender, age, educational attainment, identity, marital status, family income, and residential location.

### Other control variables

Drug utilization was measured with the following questions: (1) Do village clinics provide drugs you need? Answers were dichotomized as No = “0” and Yes = “1”; (2) What do you think of the drug effects of the village clinics? Answers were “bad, good.” Satisfaction of Rural Cooperative Medicine Insurance (RCMI) was measured with the following questions: (1) What do you think of the minimum payment of RCMI? Answers were “low, suitable, high”; (2) How about your medical expenditure after the implementation of RCMI? Answers were “decreased, unchanged, increased.”

## Statistical analysis

In order to verify the relationship between patient-doctor communication and their trust in doctors, the following strategies of statistical analysis were employed. First, descriptive statistics were used to determine the main characteristics of the samples. Second, *t*-tests were used to test the differences between the effects of different groups of contract service on trust and perception of the doctor’s communication skills. Third, Multivariate Analysis of Variance (MANOVA) was used to examine whether the differences existed in different levels among sociodemographic characteristics. Fourth, Multiple Linear Regression (MLR) was used to examine the association between contract service, doctor communication skills, and patient trust in doctors in rural clinics. According to Baron and Kenny [28], a perfect mediation is demonstrated when (a) the independent variable is significantly related to the dependent variable, (b) the independent variables are significantly related to the mediator, (c) the mediator is significantly related to the dependent variable when the independent variable is controlled. If in step (c) the previous significant relationship between independent variable and dependent variable is insignificant when the mediator is added, the effect of the independent variable on the dependent variable is completely mediated by the mediator; if its effect is still significant but its standardized regression coefficient is smaller than in the step (b), its effect is partially mediated by mediator. The regression model can be written as follows, and the Stata software package was used to implement it (version SE15, Stata Corp., College Station, TX, USA); *p*-values < 0.05 were considered statistically significant.

a $$ \mathrm{T}=\mathrm{a}+\mathrm{bCS}+{\mathrm{e}}_1 $$

b $$ \mathrm{DCS}=\mathrm{c}+\mathrm{dCS}+{\mathrm{e}}_2 $$

c $$ \mathrm{T}=\mathrm{f}+\mathrm{gCS}+\mathrm{hDCS}+{\mathrm{e}}_3 $$

In this model, *T*, *CS*, and *DCS* are the dependent variable (trust), independent variable (contract service), and mediator (doctor communication skills). The constants *a*, *c*, and *f* are the intercepts; *b*, *c*, and *h* are the regression coefficients; and *e*_*1*_, *e*_*2*_, and *e*_*3*_ are error terms.

The regression presented a direct observation of the mediating effects of doctor communication skills on the relationship between contract service and patient trust in the doctor, but it did not provide robust estimates of the goodness-of-fit between the data and the hypothesized pathway. To further confirm the hypothesis, structural equation modeling (SEM) was used as implemented by SPSS Mplus 7.0 (IBM SPSS; SPSS Inc., Armokn, NY, USA). It not only provided the standardized estimates and their standard errors for the hypothesis, but also presented goodness-of-fit indices. Two-tailed *p*-values less than 0.05 were considered statistically significant.

## Results

### Demographic characteristics

As shown in Table 1, more than 87% of the participants were over 40 years old, and more than 46% were in the age range of 40 to 60 years. Of the participants, 90.42% were married and 9.58% were single (unmarried, divorced, or widowed). As for gender, the number of males was slightly higher than that of females. A large number of participants (91.64%) identified themselves as farmers. When it came to educational background, more than half of the participants had a primary or middle school education or were even illiterate. In terms of income, 50.52% of the participants had an annual family income under 30,000RMB, while the remaining 19.34% had an annual income over 30,000RMB. Most patients suffered from hypertension and diabetes. 64.81% of the participants had medicine expenditures under 3000RMB. All participants were enrolled in Rural Cooperative Medical Insurance (RCMI), and more than half of them were satisfied with the RCMI. Turning to the clinic drug supply, two-thirds of them were satisfied. When it came to contract service, 62.37% had not signed.

**Table 1:** Sample Characteristics

Variables	*N* (574)	Percentage (%)
Gender		
Male	315	54.88
Female	259	45.12
Identity		
Farmer	526	91.64
Non-farmer	48	8.36
Age		
≤ 40	72	12.54
41–59	264	45.99
≥ 60	238	41.46
Marital status		
Married	519	90.42
Unmarried	12	2.09
Divorced or Widowed	43	7.49
Education		
Primary school or below	184	32.06
Junior high school	254	44.25
Senior high school or above	136	23.69
Family income (Yuan)		
≤ 9999	173	30.14
10,000–29,999	290	50.52
≥ 30,000	111	19.34
Type of disease		
Influenza	70	12.19
Stomach illness	50	8.70
Headache	63	10.98
Tooth disease	63	10.98
Hypertension	110	19.16
Diabetes	102	17.78
Chronic bronchitis	50	8.71
Others	66	11.50
Medicine expenditure in one year (Yuan)		
≤ 999	203	35.37
1000–2999	169	29.44
3000–5999	97	16.90
≥ 6000	105	18.29
Attitude toward minimum payment of RCMI		
Low	69	12.02
Suitable	267	46.52
High	238	41.46
Attitude toward medicine expenditure after the RCMI		
Decrease	329	57.32
Unchanged	125	21.78
Increase	120	20.91
Satisfaction with drug supply of village clinic		
Yes	384	66.90
No	190	33.10
Satisfaction with drug treatment effect		
Bad	19	3.31
Good	555	96.69
Status of contract service		
Signed	358	62.37
No	216	37.63

### Patient-doctor communication

Table 2 summarizes the description of the patient perceptions of doctors’ therapeutic communication and patients’ communication confidence. As indicated in Table 2, patients’ scores on each of the separate items were high. Their average communication scores were 5.72 and 3.83 (*SD* = 1.40; range:1–7).

**Table 2:** Description of TCom-skills GP scale

Items	Obs	Mean	*SD*	Min	Max
1. Does the general practitioner take time to listen to me?	574	5.90	1.36	1	7
2. Does everything make me feel I can trust him/her?	574	5.50	1.32	1	7
3. Does the general practitioner explain what the treatment is for?	574	5.62	1.38	1	7
4. Does the general practitioner take account of my preferences in prescribing medication?	574	5.68	1.41	1	7
5. Does the general practitioner give me the impression he/she has respect for me?	574	5.89	1.54	1	7
6. Does the general practitioner give me information on the side effects of medication?	574	5.65	1.48	1	7
7. Does the general practitioner emphasize which are the most important drugs?	574	6.00	1.11	1	7
8. Does the general practitioner discuss any difficulties I have in complying with the treatment?	574	5.06	1.82	1	7
9. Does the general practitioner explain things in simple words?	574	5.71	1.43	1	7
10. Does the general practitioner offer new treatments?	574	5.58	1.49	1	7
11. Does the general practitioner write the prescription legibly?	574	5.43	1.62	1	7
12. Does the general practitioner let me ask questions?	574	5.91	1.33	1	7
13. Does the general practitioner give me incentives to comply with the treatment?	574	5.90	1.29	1	7
14. Does the general practitioner give me advice on prevention (diet, physical activity)?	574	6.05	1.18	1	7
15. Does the general practitioner give the impression he/she knows his/her job?	574	5.93	1.31	1	7
Total score		5.72	1.40	1	7

### Contract service

A comparison was made of the demographic characteristics between those who had signed or not signed up for the contract service. As shown in Table 3, there were no significant demographic differences between the two groups. Table 4 shows the result of the characteristics of contract service and *t*-tests on the main variables. The results show that patients in Dafeng signed the most contract services among the three areas, while Yinan had the most patients who did not sign up for the contract service. Across the three areas, 358 patients signed up for the contract service and 216 did not. Comparison between the two groups showed a significant difference (*p* ≤ 0.001). As shown in Table 4, patients who signed the contract presented higher scores for trust and communication skills (mean scores = 3.59, 5,85) than did those who did not sign (mean scores = 3.02, 5.11).

**Table 3:** The comparison of demographic characteristics between those who signed and did not sign up for contract service

Variables	Signed (*N* = 358)	Not signed (*N* = 216)	*p*-value
Gender	1.15 ± 0.05	1.14 ± 0.49	*t =* −1.47, *p* = 0.93
Identity	1.07 ± 0.27	1.09 ± 0.29	*t* = 0.60, *p* = 0.27
Age	2.32 ± 0.67	2.24 ± 0.68	*t* = − 1,33, *p* = 0.91
Education status	1.94 ± 0.75	1.88 ± 0.73	*t* = − 1.04, *p* = 0.85
Marital status	1.20 ± 0.58	1.13 ± 0.47	*t* = − 1.42, *p* = 0.92
Family income (Yuan)	1.88 ± 0.71	1.91 ± 0.67	*t* = 0.54, *p* = 0.30
Medicine expenditure in one year	2.23 ± 1.08	2.09 ± 1.14	*t* = − 1.49, *p* = 0.93

**Table 4:** Contract service characteristics and comparison of the two groups

	Signed			Not signed				
	Freq.	Percent	Mean	Freq.	Percent	Mean	Mean-diff.	*t*
Dafeng	138	78.41		38	25.19			
Yinan	96	47.76		105	52.24			
Wufeng	124	62.94		73	37.06			
Trust	358		3.59	216		3.02	−0.17***	−3.93
Communication skills	358		5.85	216		5.11	−0.35***	−4.14

*** *p* ≤ 0.001

### Effects of demographic variables on trust

MANOVA was employed (see Table 5) to test whether there were significant demographic differences on patients’ trust in doctors.^1^ The results showed that patients’ gender, identity, education status, and family income did not lead to significant differences.

**Table 5:** Comparison of the effects of the demographic variables on trust

Variables	Partial *SS*	*DF*	*f*	*p-*value
Gender	0.00	1	0.98	0.321
Identity	0.61	1	7.00	0.008**
Age	2.84	2	0.38	0.687
Marital status	1.88	2	5.35	0.005**
Education status	0.39	2	0.40	0.669
Family income (Yuan)	0.02	2	2.91	0.056
Medicine expenditure in one year	0.80	3	1.04	0.374

** 0.001 < *p* ≤ 0.01

### Correlations between contract service, doctor communication skills, and trust

The correlations between contract service, doctor communication skills, and trust were analyzed using pairwise Pearson’s correlation (see Table 6). The results showed that contract service was significantly correlated with trust (*p* ≤ 0.01) and doctor communication skills (*p* ≤ 0.001), while doctor communication skills were significantly correlated with trust (*p* ≤ 0.001).

**Table 6:** Pairwise correlation between contract service, doctor communication skills, and trust

	Trust	Doctor communication skills	Contract service
Trust	1		
Doctor communication skills	0.40^***^	1	
Contract service	0.16^**^	0.17^***^	1

** 0.001 < *p* ≤ 0.01, *** *p* ≤ 0.001

### The mediating role of doctor communication skills

**Fig. 1:**
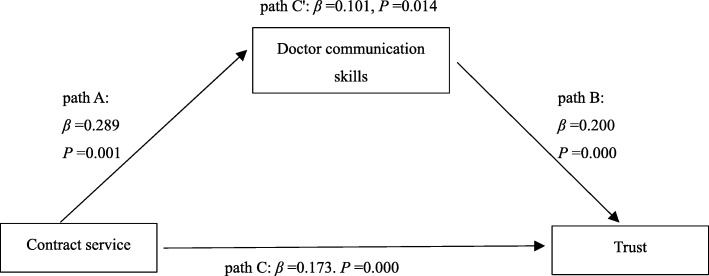
Doctors communication skills as a mediator between contract service and trust. Note: Path A: the IV (contract service) significant influences the DV (trust) in the absence of the mediator (doctor communication skills). Path B: the mediator (doctor communication skills) has a significant influence on DV (trust) and also means that the direct effect of doctor communication skills on the DV (trust). Path C: IV (contract service) significant influence the DV (trust). Path C′: the IV (contract service) significant influences the DV (trust) on the addition of the mediator (doctor communication skills). Model goodness-of-fit statistics: χ^2^ = 12.26, DF = 3, CFI = 0.99, TLI = 0.99, RMSEA = 0.05

Table 7 and Fig. 1 summarize the regression results for the pathway from contract service to patient trust in doctor through doctor communication skills. The results show that contract service had both a direct and an indirect influence on patient trust in doctors. As shown in Fig. 1, Path A represents the direct effect of contract service on doctor communication skills (β = 0.289, *p* < 0.05); Path B represents the direct effect of doctor communication skills on patient trust in doctor (β = 0.200, *p* < 0.05); Path C represents the direct effect of contract service on patient trust in doctor (*β* = 0.173. *p* < 0.05); and Path C′ represents the coeffect of contract service and doctor communication skills on patient trust in doctor (β = 0.101, *p* < 0.05). The goodness-of-fit indices (*χ*^2^ = 12.26, *DF* = 3), Comparative Fit Index (CFI = 0.99), Tucker-Lewis Index (TLI = 0.99), and Root Mean Square Error of Approximation (RMSEA = 0.05) indicate a strong fit between the observed values and expected values.

**Table 7:** The mediating role of doctor communication skills

	Path A	Path B	Path C	Path C′
	Direct effects of contract service on doctor communication skills	Direct effects of doctor communication skills on trust	Direct effects of contact service on trust	Coeffect of doctor communication skills and contract service
Contract service	0.289^***^		0.173^***^	0.101^**^
Doctor communication skills		0.200^***^		0.190^***^
_−_Cons	5.203^***^	1.997^***^	3.039^***^	2.047^***^
Adjust-*R*^2^	0.104	0.173	0.074	0.180
*p*	0.001	0.000	0.000	0.000

** 0.001 < *p* ≤ 0.01, *** *p* ≤ 0.001

## Discussion

To our knowledge, this study may be the first to explore the potentially important mediational pathway between contract service and rural patient trust in village doctors in the village clinics of rural China.

In this study, we have identified contract service as an independent predictor of rural patient trust in their doctors after controlling for other factors (β = 0.173, *p* < 0.01), which confirmed the hypotheses we set up. A previous study explained that contract service made patients satisfied in their relationships with village doctors because they enjoyed the service of public health that they felt they deserved [12]. This study confirmed that contract services contributed to the improvement of the patient-doctor relationship and their trust-building. For one thing, the contract service increased doctors’ annual income, which improved their job-related efficiency and positivity; for another, it changed the service manner of village doctors, who previously sat passively in clinics waiting for patients to visit, while after signing the contract service, doctors must provide service actively, as by home visits and follow-ups [12, 29]. An earlier study confirmed that contract services increased the work burden of village doctors and brought about staff shortages when providing public health and primary prevention [12], but another study analyzed the contract service as increasing convenience and patient safety and alleviating their uncertainties on health [30]. Other studies have also confirmed that contract service routinized the professional and technique training and enhanced the service capability of village doctors [12, 29]. The findings of this study are consistent with previous studies indicating that contract patients had a positive perception of doctors’ communication skills during the provision of services. A recent study discussed the differences between patients who had and had not signed up for contract service on their reasons for accepting the services, which indicated that patients who signed the contract were more likely to accept the services than those who had not signed [31]. Similarly, this study also found that patients who had and had not signed the contract service presented significant differences in their perceptions of doctor communication skills. Patients who had signed up for the contract service showed a higher perception of doctor communication skills than those who had not.

The most significant finding of this study is the discovery of the mediating effect of doctors’ communication skills between contract service and patients’ trust in village doctors in the village clinics of rural China. The result showed that the significant influence of contract service in the model decreased when the variable of doctor communication skills was added (*p* = 0.014). Numerous studies have verified that communication skills of doctors influence the patient-doctor relationship among a variety of patients in different cultural environments [32–34]. The results of this study together with overseas research further confirm the importance of doctor communication skills in patient-doctor trust. A study by Jennifer Fong Ha et al. determined that doctors expressing a willingness to listen increased patients’ confidence and willingness to talk, and their patients thus generally shared more information about their symptoms [35]. This in turn helps doctors carry out causal analyses and provide appropriate treatment [36]. Previous studies showed that most deterioration in the patient-doctor relationship was due to the inadequate explanation of the diagnosis or treatment [37, 38] and to patients feeling ignored [39]. However, this study found that the mean score of doctor communication skills (Table 2) was high, which indicates that village doctors used good communication skills in their daily work. For instance, the ability to clearly explain prescriptions reduces confusion and misunderstanding among patients taking medication [34]. Doctors who explain why they are prescribing a particular drug or choosing one treatment over another increase their patients’ ability to understand prescription choices; they reduce the levels of anxiety, worry, and fear of illness among those patients. Clear explanations of drugs and medications, including both positive and negative efficiency, reduce fear and uncertainty and increase the likelihood that patients will comply with their doctors’ advice [35]. Finally, these communication skills served to increase patients’ trust in their doctors.

## Limitations

This is a preliminary and exploratory study, and the present findings require further investigation. This study has several limitations. First, we only chose three counties in the eastern and central parts of China as our study sites according to economy and region, and even though the study was sufficient to support the statistical analysis we used, its results cannot be extrapolated to the whole country. Further research is needed to expand the sample size when analyzing the complex dynamic mechanism between contract service and patients’ trust in doctors. Second, although we adjusted for some potential confounders likely to have an impact on patients’ trust in doctors in the multiple regression analysis, other confounders may have been overlooked, such as the rural interpersonal relationship, which should be considered in future studies. Third, this study used a questionnaire methodology to collect data, which might suffer the social-desirability bias effect because of the lack of observation data. Last but not least, this cross-sectional data cannot be used to analyze the direction of causality. Future studies will need to improve the sampling and use a longitudinal study design, a comprehensive questionnaire, observations, and perhaps even experimental intervention to test and extend this research.

Despite these limitations, this study is the first to use the TCom-skills GP scale and WFPTS to measure village doctors’ communication skills and patients’ trust in doctors to explore trust-building in China’s rural culture.

## Conclusions

This study empirically investigated the relationship between the policy of contract service, a doctor’s communication skills, and patient-doctor trust in the village clinics of rural China. Contract service plays a vital beneficial role in building patient-doctor trust. Doctors’ therapeutic communication skills played a mediating role between contract service and patients’ trust in doctors in that adequate patient-doctor communication increased patients’ trust in their doctors.

## Abbreviations

GP: General Practitioner
MLR: Multiple Linear Regression
RCMI: Rural Cooperative Medicine Insurance
TCom-skills: Therapeutic Communication Skills
WFPTS: Wake Forest Physician Trust Scale

## Footnotes

1 Considering that the role of MANOVA was to test the differences in the effects of the demographic variables on patient trust in doctor, we only considered the main effect of the variable, and disregarded the interaction effects.
